# Supplementary material for: Comparison of Storage-Related Volatile Profiles and Sensory Properties of Cookies Containing Xylitol or Sucrose
Source: Foods. 2023 Nov 26;12(23):4270. doi: 10.3390/foods12234270 (PMC10706587; doi:10.3390/foods12234270)
Supplement: Supplementary file 1 [file foods-12-04270-s001.zip › foods-2680973-supplementary.pdf]

Supplementary

Article

Comparison of storage-related volatile profiles and sensory properties of cookies containing xylitol or sucrose

Jaroslawa Rutkowska *, Damian Baranowski, Agata Antoniewska-Krzeska and Eliza Kostyra

Institute of Human Nutrition Sciences, Faculty of Human Nutrition, Warsaw University of Life Sciences, Nowoursynowska St. 159c, 02-776 Warsaw, Poland; damian_baranowski@sggw.edu.pl (D.B.); agata_antoniewska@sggw.edu.pl (A.A.-K.); eliza_kostyra@sggw.edu.pl (E.K.)

* Correspondence: Correspondence: jaroslawa_rutkowska@sggw.edu.pl

Table S1: Sensory attributes and definitions created by the panel members for Quantitative Descriptive Analysis

Attribute	Definition
**Appearance**	
Color (light yellow color - brown color)	Brown color intensity on the cookie surface, between light-yellow and brown.
**Aroma**	
Buttery (not detected - extremely intense)	Fresh butter aroma.
Sweet/Caramelized (not detected - extremely intense)	Sweet, aromatic sensation typical of the burnt sugar.
Fruity (not detected - extremely intense)	Sweet aroma which evokes impression of fruit.
Nutty (not detected - extremely intense)	Specific odor of walnuts and hazelnuts.
Roasty (not detected - extremely intense)	Aroma associated with freshly baked shortbread cookies or bread.
Rancid (not detected - extremely intense)	Aroma associated with stored vegetable oil.
Sour (not detected - extremely intense)	Vinegar aroma.
Pungent (not detected - extremely intense)	Strong odor which causes unpleasant, sharp or irritating sensation.
**Taste**	
Buttery (not detected - extremely intense)	Fresh butter taste.
Sweet (not detected - extremely intense)	Typical taste of sucrose.
Roasty (not detected - extremely intense)	Taste associated with freshly baked shortbread cookies or bread.
Bitter (not detected - extremely intense)	Typical taste of caffeine or quinine.
Aftertaste - cooling effect (not detected - extremely intense)	Cooling in-mouth sensation related with dissolving sweetener in saliva.
**Texture**	
Hardness (soft - hard)	Minimum force necessary to bite the sample.
Fracturability (very weak - strong)	Ease of breaking off a piece of cookie with teeth.
Moisture (very dry - very moist)	Perceived amount of water in the cookie.
Adhesiveness (very weak - strong)	Necessary force to completely remove the sample from the palate using tongue.
